# Congenital depressed skull fracture in a neonate without obstetric trauma

**DOI:** 10.4102/sajr.v30i1.3305

**Published:** 2026-02-05

**Authors:** Elliot K. Mmutle, Baby S. Lekhuleni, Luvo Gaxa

**Affiliations:** 1 Department of Radiology, Witbank Tertiary Hospital, Emalahleni, South Africa

**Keywords:** congenital depressed skull fracture, neonate, non-traumatic skull depression, neonatal head injury, ping pong, CT imaging, uncomplicated delivery, paediatric imaging

## Abstract

Congenital depressed skull fractures (ping-pong fractures) without obstetric trauma are rare. A term male neonate delivered via uncomplicated caesarean section, demonstrated a right parieto-temporal skull depression (5 cm × 5 cm) at birth. Computed tomography revealed a 4 mm parietal depression without intracranial injury. No instrumental delivery or maternal trauma were present. The likely aetiology was intrauterine compression (‘faulty foetal packing’). The patient was managed conservatively with close follow-up.

**Contribution:**

This case underscores the importance of perinatal history and neuroimaging in distinguishing spontaneous from traumatic fractures and supports conservative management in neurologically intact infants.

## Introduction

Neonatal depressed skull fractures, or ping-pong fractures, are inward deformities of the calvarium without cortical disruption.^1^ They occur in 1–2.5 per 10 000 live births.^2,3^ While most result from obstetric trauma, spontaneous congenital cases without identifiable risk factors are infrequent. They may be caused by intrauterine compression from maternal or foetal structures – a mechanism termed ‘faulty foetal packing’.^4,5^ The key clinical concern is a possible underlying brain injury.^6^

## Patient presentation

A 2.6 kg male neonate was born at 38 weeks to a healthy 35-year-old woman (G3P3). The pregnancy was uncomplicated. The mother had prolonged rupture of membranes and failed induction of labour at 38 weeks of gestational age. After failed induction, an uncomplicated caesarean section was performed without the use of extraction instruments.

**FIGURE 1:**
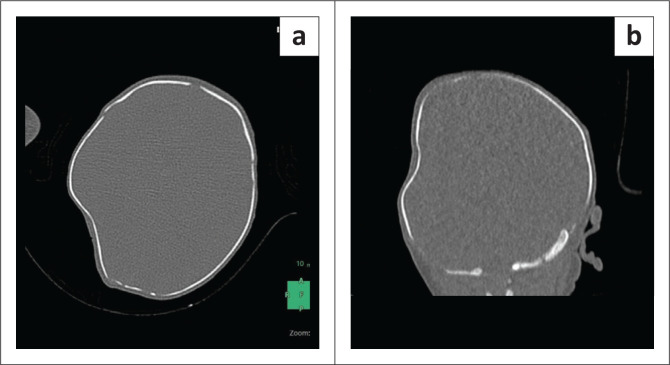
(a) Axial and (b) coronal view of the CT brain scan (bone window).

Apgar scores were 9/10, 10/10, and 10/10 at birth at 1 min, 5 min, and 10 min, respectively. Physical examination revealed a non-tender, 5 cm × 5 cm right parieto-temporal indentation, without oedema or bruising (Figure 1a). Neurological examination was normal.

Non-contrast CT demonstrated a smooth inward deformation of the right parietal bone, measuring approximately 4 mm in depth, without a fracture line, intracranial haemorrhage, or cerebral oedema on axial and coronal images (Figure 1a and Figure 1b). Given the absence of trauma, instrumental delivery, or maternal abdominal injury, the diagnosis was a congenital depressed skull fracture likely because of intrauterine compression. The patient was managed conservatively with serial neurological assessments, and follow-up imaging was planned.

## Discussion and literature review

Neonatal depressed skull fractures – commonly termed ping-pong fractures – are inward concavities of the neonatal calvarium without complete bone discontinuity, resembling the indented surface of a ping-pong ball.^1^ In series and population studies, the reported incidence is low, generally quoted in the range of 1.0–2.5 per 10 000 live births, although estimates vary by cohort and reporting practice.^1,2,3^

Two overlapping but distinct concepts are used in the literature: (1) true ping-pong (depressed) skull fracture – often associated with perinatal trauma (e.g. forceps or vacuum), and (2) congenital vault depression or faulty foetal packing, a non-traumatic, in-utero concavity caused by persistent localised pressure on the pliable foetal skull. Clinically and radiologically, the lesions can be indistinguishable; differentiation often rests on perinatal history and the presence or absence of soft-tissue injury or other trauma markers.^4,5,6^

The neonatal skull is unusually malleable because of incomplete ossification and a thin cortical layer; localised, sustained pressure can produce an inward deformity without a cortical fracture. Reported intrauterine pressure sources include maternal bony prominences (sacral promontory, symphysis pubis), uterine fibroids, scoliosis or pelvic asymmetry, twin or foetal parts against the skull, and abnormal foetal lie – collectively described in older and more recent reports as mechanisms of ‘faulty foetal packing’.^4,7,8,9,10^ Traumatic ping-pong fractures occur when external force through difficult labour or instrumentation (forceps, vacuum, prolonged labour) indents, but does not fracture the pliable skull.^3,7^

Most neonates with congenital depressed skull deformities are otherwise well at birth: normal Apgar scores, absence of neurological signs, and no overlying soft-tissue haematoma, and by contrast, traumatic cases may have scalp laceration, cephalohaematoma, or other signs of delivery trauma.^6,10,11^ Neurological deficit or signs of raised intracranial pressure are uncommon in uncomplicated congenital depressions, but mandate urgent imaging and neurosurgical input if present.^6,11^

**FIGURE 2:**
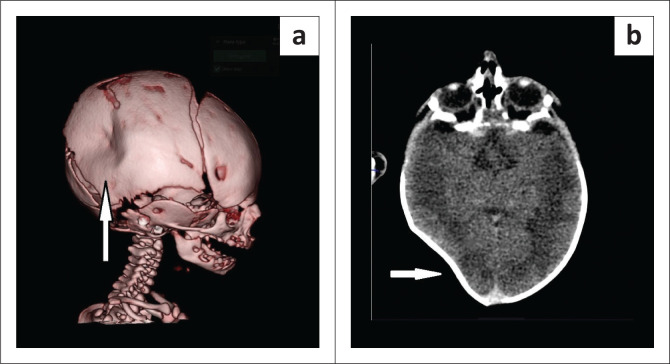
(a) Three-dimensional volume-rendered CT reconstruction demonstrates a focal depressed deformity (white arrow) of the right parieto-temporal skull without cortical discontinuity, and (b) axial CT image (soft tissue window) shows the corresponding inward depression (white arrow) of the calvarium.

Plain radiography can identify contour depression but has limited sensitivity for intracranial sequelae. Non-contrast CT is the imaging examination of choice for acute assessment, as it defines the depth and shape of the depression, detects linear fracture lines, extradural, subdural, or intracerebral haemorrhage, and mass effect. Three-dimensional volume rendering is useful for surgical planning or clear pictorial demonstration of the deformity.^9^ Radiologically, both congenital and traumatic forms appear as smooth inward deformities without cortical breach, but differentiation requires a detailed perinatal history, as imaging alone may be equivocal.^6,9^ The absence of overlying soft tissue injury or neurological deficits favours a congenital origin. Three-dimensional reconstruction further illustrated the focal parieto-temporal skull depression without associated intracranial abnormality (Figure 2a), with corresponding axial soft tissue images confirming the absence of extracranial or intracranial injury (Figure 2b).

Key differential diagnoses include traumatic ping-pong fracture, congenital vault depression/faulty foetal packing, cranial meningocele or encephalocele (would show intracranial herniation on imaging), and externally applied moulding from delivery (transient moulding usually resolves). Management depends on neurological status and imaging. Conservative management is recommended in neurologically intact neonates with isolated depression and no intracranial pathology.^5,11^ Because of rapid brain growth, the skull often remodels spontaneously within weeks to months. Manual or suction-assisted elevation and surgical correction are reserved for cases with mass effect, intracranial injury, or significant cosmetic deformity.^7,10^

Given the condition’s rarity, there is no standardised management algorithm; decisions should be individualised and guided by multidisciplinary assessment involving neonatology, radiology, neurosurgery, and obstetrics. Detailed documentation of the clinical course and radiological findings facilitates optimal care and contributes to the limited body of literature, thereby improving diagnostic awareness and management strategies. Long-term follow-up is warranted to assess cranial contour, neurological development, and potential late sequelae. Broader multicentre case registries and prospective studies are needed to clarify the incidence, refine treatment criteria, and optimise outcomes for this rare, but crucial neonatal presentation. This case adds to the limited literature describing congenital depressed skull fractures without identifiable risk factors. Awareness of this entity can prevent unnecessary intervention.

## Conclusion

Congenital depressed skull fracture in the neonate is an uncommon clinical entity that presents in the absence of identifiable obstetric trauma or instrumental delivery. Although its radiological and clinical appearance may closely mimic traumatic ping-pong fractures, careful perinatal history, thorough physical examination, and targeted imaging are essential to distinguish between congenital and acquired causes. Current evidence suggests that most neurologically intact neonates with isolated, shallow depressions and no associated intracranial injury achieve spontaneous remodelling with conservative management. At the same time, closed suction elevation offers a minimally invasive corrective option for persistent or cosmetically significant deformities. Surgical intervention remains reserved for cases with neurological compromise.

## References

[1] Ben-Ari Y, Merlob P, Hirsch M, Reisner S. Congenital depression of the neonatal skull. Eur J Obstet Gynecol Reprod Biol. 1986;22(4):249–255. 10.1016/0028-2243(86)90073-0 3743864

[2] Mehmet S, Ramazan F, Bora G, Hayri K, Zeki S. Spontaneous elevation of a ping-pong fracture: Case report and review of the literature. Pediatr Neurosurg. 2013;48(5):324–326. 10.1159/000351412 23796696

[3] Abbassioun K, Amirjamshidi A, Rahimizadeh A. Spontaneous intrauterine depressed skull fractures. Child’s Nerv Syst. 1986;2:153–156. 10.1007/bf00270846 3779672

[4] Whitby EH, Griffiths PD, Rutter S, et al. Frequency and natural history of intracranial haemorrhage in term neonates. Lancet. 2004;9442(364):1365–1367. 10.1016/s0140-6736(04)15730-9

[5] Govaert P, Vanhaesebrouck P, De Praeter C. Vacuum extraction, bone injury and neonatal subgaleal bleeding. Eur J Pediatr. 1992;151:532–535. 10.1007/bf01957762 1396918

[6] Tovar-Spinoza Z, Kim PD. Congenital depressed skull fracture in the absence of trauma: Case report and literature review. Res Rep Neonatol. 2012;2:11–14. 10.2147/rrn.s29666

[7] De Paul D, Njamnshi AK, Ongolo-Zogo P, Ako S, Essomba A, Sosso MA. Depressed skull fractures in children: Treatment using an obstetrical vacuum extractor. Pediatr Neurosurg. 2006;42(5):273–276. https://karger.com/pne/article-abstract/42/5/273/276533/Depressed-Skull-Fractures-in-Children-Treatment?redirectedFrom=fulltext 16902337 10.1159/000094061

[8] Robertson RL, Robson CD, Zurakowski D. CT versus MR in neonatal brain imaging at term. Pediatr Radiol. 2003;(33):442–449. 10.1007/s00247-003-0933-6 12743660

[9] Dharmaraj ST, Embleton ND, Jenkins A. Depressed skull fracture in a newborn baby. Arch Dis Child Fetal Neonatal Ed. 2009;94:F137. 10.1136/adc.2008.148718 19240294

[10] Ilhan O, Bor M, Yukkaldiran P. Spontaneous resolution of a ‘ping-pong’ fracture at birth. BMJ Case Rep. 2018;2018:bcr-2018-226264. 10.1136/bcr-2018-226264 PMC6157499 30249736

[11] Hanlon L, Hogan B, Corcoran D, Ryan S. Congenital depression of the neonatal skull: A self-limiting condition. Arch Dis Child Fetal Neonatal Ed. 2006;91(4):F272. 10.1136/adc.2005.082347 16790730 PMC2672729

